# Relationship between adiponectin and blood pressure in obese Latino adolescent boys with a family history of type 2 diabetes

**DOI:** 10.1186/s12887-023-04337-y

**Published:** 2023-10-20

**Authors:** Kristin Hijazin, Brandon Smith, Coleby Garrett, Allan Knox, Louise A. Kelly

**Affiliations:** Department of Exercise Science, California Lutheran University, 60 W. Olsen Road, #3400, Thousand Oaks, CA 91360 USA

**Keywords:** Latino youth, Pediatric, Males, Adiponectin, Blood pressure, Obesity

## Abstract

**introduction:**

Adipokines are associated with several pathological states including, metabolic syndrome, obesity, insulin resistance and type 2 diabetes. One of these adipokines, adiponectin is of particular interest as it has been shown to have numerous anti-inflammatory effects, However, the association between adiponectin and blood pressure remains inconclusive especially in the Latino adolescent with obesity.

**Purpose:**

To investigate the relationship between plasma adiponectin and blood pressure in Latino adolescents’ boys with obesity and a with a family history of Type 2 diabetes.

**Methods:**

Thirty two Latino adolescent males with obesity aged 14–17 years with a family history of type 2 diabetes underwent a frequently sampled glucose tolerance test (FSIVGTT) to measure insulin sensitivity. Body composition was assessed using dual energy x-ray absorptiometry. Obesity was defined as having a BMI percentile ≥95. Blood pressure was assessed using the Dinamap automated blood pressure monitor, and the average of three readings was used in the analysis. Fasting plasma adiponectin was determined using radioimmunoassay.

**Results:**

There were moderate positive significant correlations for adiponectin and Systolic blood pressure(SBP) (rho = 0.436, p < 0.027) and Diastolic blood pressure(DBP) (rho = 0.41,p < 0.028). A multivariate liner regression showed that plasma adiponectin could significantly detect 19% of the variance in SBP (p = 0.017, and 33% for DBP (p = 0.017). In a simple linear regression adiponectin was not related to any of our variables (p < 0.05).

**Conclusion:**

In conclusion, adiponectin was positively and significantly correlated to blood pressure in Latino adolescent with obesity. Future studies should investigate this relationship in a large sample of Latino adolescent youth.

**What is known about this subject**:

The prevalence of hypertension in children is threefold higher in obese than non-obese children. Furthermore, the cytokine adiponectin has been associated with the regulation of blood pressure (BP) in adults and adolescents. Adiponectin has been shown to be associated with Type 2 diabetes and the metabolic syndrome.

**What this study adds**:

Adiponectin was positively and significantly correlated to blood pressure in obese Latino adolescent youth. To our knowledge, we are the first group to investigate the relationship between adiponectin and blood pressure in obese Latino adolescent boys with a family history of type 2 diabetes.

## Introduction

Pediatric obesity is one of the most pertinent health issues of the last century. In 2019, 340 million children aged 5–19 years are considered overweight/obese [1, 2] with a disproportionate impact in racial and ethnic minorities, particularly Latino youth [3]. The 2011–2020 National Health and Nutrition Examination Survey (NHANES) shows an increased trend of obesity (95th percentile for age and gender) in Latino youth when compared to other ethnic groups of the same age from 21.8 to 27.0%; P for trend = 0.006 [4]. Studies have also shown that the prevalence of obesity is significantly higher in Latino males. Furthermore, pediatric obesity is commonly associated with several non-communicable diseases such as type 2 diabetes [5], metabolic syndrome [6], fatty liver disease (7), cardiovascular disease [8], several cancers [9] and hypertension [9–12].

Low insulin sensitivity (S) is also a detector of high blood pressure (BP), particularly in minority youth [13]. In adult and pediatric studies, hypertension has been associated with insulin resistance and hyperinsulinemia [12, 13]. In a meta-analysis by Wang and colleagues the relationship between insulin resistance and hypertension was investigated in 11 studies, involving 10,230 participants. The results of this analysis suggested that insulin resistance is independently associate with hypertension in the general population [14].

The prevalence of hypertension in children is threefold higher in obese children compared to non-obese children [12]. A systemic review of 22 articles by Lona et al., concluded children with higher BP’s and BMIs have increased cardiovascular biomarker central pulse wave velocity (cPWV) [15]. A higher blood pressure (BP) is also associated with a higher body mass index (BMI) [16], both of which are higher in children from minority backgrounds compared with their white counterparts [17]. In addition, changes in secretion of cytokines such as adiponectin have been linked to cardiovascular risk factors through the effects on insulin sensitivity.

Furthermore, adiponectin, an adipose tissue-derived protein with insulin-sensitizing and antiatherogenic properties, has been associated with the regulation of BP in adults and adolescents [18, 19]. During puberty, adiponectin has been shown to decrease in boys [20] and is associated with Type 2 diabetes [21] and metabolic syndrome [22]. Therefore, the aim of this study was to investigate the relationship between plasma adiponectin and blood pressure in Latino adolescent boys with obesity and a family history of Type 2 diabetes.

## Methods and procedures

### Participants

Forty-three (*N* = 43) participants were recruited from the greater Los Angeles County area through medical clinics, advertisements, and local schools to participate in the study (Families United for Education and Research for Strong Adolescent Latinos, **FUERSA**). Participants were recruited to the study if they met the following study inclusion criteria: (1) male; (2) grades 9th through 12th (approximately 14–18 years of age); (3) with a BMI ≥95th percentile for age and sex; (4) of Latino ancestry (parents and grandparents descent as determined by self-report); (5) absence of diabetes using established guidelines; (6) absence of comorbid inflammatory disease, secondary hypertension or any condition that would predispose them to type 2 diabetes;7) have a positive family history of type 2 diabetes (1st degree which was determined by parental self-report). The study was conducted in accordance with the guidelines of the Helsinki Declaration. Written informed consent and assent were obtained from the parents and children prior to testing. The Institutional Review Board of the University of Southern California approved the study.

### Anthropometric measures and body composition

Height was measured with a stadiometer to the nearest 0.1 cm. Body mass was measured without shoes and in a hospital gown to the nearest 0.05 kg using a beam medical scale. Body mass index (BMI) was calculated; age- and sex-specific BMI percentile were determined using *EpiInfo 2000, Version 1.1* (CDC, Atlanta, GA). Obesity was defined as have a BMI ≥95th percentile for age and sex. A dual-energy X-ray absorptiometry (DEXA) scan (Hologic QDR 4500 W; Bedford, MA) was performed to estimate total fat mass (FM) and total lean tissue mass (LTM).

### Blood pressure measurement

Resting systolic blood pressure (SBP) and diastolic blood pressure (DBP) were measured in the sitting position using a Dinamap automated blood pressure monitor (Critikon Inc., Tampa, FL) with the arm supported at heart level. After sitting quietly for 5 min, measurements were obtained on each child using an appropriately sized cuff placed on the right arm. Three readings of blood pressure were obtained, and the average was recorded, according to the recommendations of the American Heart Association [23]. Systolic minus diastolic was used to calculate pulse pressure.

### Blood sampling and analysis

A venous blood sample was taken after 12 h overnight of fasting for the following measurements, plasma glucose, and insulin as previously described [24, 25]. The insulin resistance index derived by frequently sampled intra venous glucose tolerance test (FSIVGT) was previously described [24, 25]. Fasting plasma adiponectin was measured in duplicate using radioimmunoassay (RIA) kits obtained from Linco Research (St. Charles, MO) following the manufacturer’s protocol. The intra- and interassay coefficients of variation were less than 10%.

### Statistical analyses

All data were checked for normality prior to statistical analysis using descriptive statistics, histograms with normal distribution curves, and the Anderson-Darling (AD) normality tests. Data are presented as means and standard deviation unless indicated otherwise. The correlation analyses in Table 1 and multivariate linear regression analyses were carried out to investigate whether plasma adiponectin could significantly detect SBP and DPB (see Tables 2 and 3). A simple liner regression was used to detect possible relationships to study variables. All analysis was conducted using SPSS (version 24 for Mac) with significance or with an alpha > set at 0.05.

**Table 1:** Correlations between Adiponectin, anthropometric, body composition, insulin resistance, fasting glucose, CRP and blood pressure

Variables	Systolic BP		Diastolic BP	
	rho	p	rho	p
Age (yr.)	-0.12	0.58	0.19	0.31
Height (m)	-0.20	0.29	-0.21	0.27
Weight (kg)	0.27	0.16	0.14	0.46
BMI (kg/m^2^)	0.26	0.18	0.18	0.35
BMI %tile (%)	-0.14	0.48	-0.19	0.31
Systolic Blood Pressure (mmHg)			0.69	0.00*
Diastolic Blood Pressure (mmHg)	0.67	0.00*		
Pulse rate (BPM)	0.03	0.97	0.36	0.075
Waist circumference (m)	-0.18	0.36	-0.23	0.23
Hip Circumference (m)	− 0.019	0.31	-0.19	0.32
Waist/hip ratio	-0.05	0.79	-0.16	0.41
Fat (kg)	-0.03	0.88	-0.09	0.65
Lean tissue (kg)	0.00	0.99	0.11	0.58
Lean Tissue and Bone Mineral Content (kg)	− 0.018	0.92	0.09	0.62
Total Mass (kg)	-0.34	0.99	0.03	0.87
Fat (%)	0.06	0.78	-0.11	0.57
Lean (%)	0.06	0.74	0.13	0.51
Insulin Sensitivity (X10^− 4^ min^− 1^/µU/ml)	0.30	0.12	0.26	0.18
Acute Insulin Response (µU/ml X 10 min)	-0.03	0.99	0.08	0.67
Disposition Index (X10^− 4^ min^− 1^)	-0.33	0.09	-0.17	0.39
Fasting Glucose (mg/dl)	0.12	0.53	0.06	0.77
CRP (ng/ml)	-0.12	0.56	-0.02	0.32
Adiponectin (µg/ml)	0.44	0.02*	0.41	0.02*

**Table 2:** Multiple linear regression analysis for SBP

	Model 1	Model 2	Model 3	Model 4	Model 5	Model 6
Adiponectin (µg/ml)	0.439± 0.061 0.022	0.472±0.060 0.013	0.462±0.063 0.021	0.488± 0.065 0.019	0.350±0.075 0.127	0.308± 0.77 0.189
BMI (kg/m2)		0.273±0.331 0.134				
Waist Circumference (cm)			-0.043±0.188 0.817			
Fasting Glucose (mg/dl)				-0.118±0.168 0.553		
Insulin Sensitivity (X10^− 4^ min^− 1^/µU/ml)					0.278±1.285 0.227	
Disposition Index (X10^− 4^ min^− 1^)						-0.236±-0.003 0.378

The regression coefficient (β±SE) and P value are indicated

**Table 3:** Multiple linear regression analysis for DPB

	Model 1	Model 2	Model 3	Model 4	Model 5	Model 6
Adiponectin (µg/ml)	0.428± 0.047 0.026	0.441±0.048 0.025	0.408±0.05 0.045	0.401±0.052 0.06	0.348±0.061 0.159	0.324± 0.064 0.021
BMI (kg.m2)		0.110±0.265 0.558				
Waist Circumference (m)			-0.130± 0.149 0.505			
Fasting Glucose (mg/dl)				0.033± 0.135 0.868		
Insulin Sensitivity (X10^− 4^ min^− 1^/µU/ml)					0.107±1.061 0.662	
Disposition Index (X10^− 4^ min^− 1^)						0.324±0.64 0.207

The regression coefficient (β±SE) and P value are indicated

## Results

Thirty-two (*N* = 32) Latino adolescent males with obesity were consented into the study. Characteristics of participants are shown in Table 4.

**Table 4:** Characteristics of Participants (n = 32)

Variables	Mean ± SD N = 32
Age (yr)	15.28 ± 1.07
Height (m)	169.47 ± 13.00
Weight (kg)	96.10 ± 15.32
BMI (kg/m^2^)	33.14 ± 4.42
BMI %tile (%)	97.61 ± 2.02
Systolic Blood Pressure (mmHg)	124.61 ± 8.34
Diastolic Blood Pressure (mmHg)	67.12 ± 6.37
Pulse rate (BPM)	70.39 ± 10.77
Waist circumference (m)	0.962 ± 8.46
Hip Circumference (m)	1.083 ± 7.17
Waist/hip ratio	0.89 ± 0.05
Fat (kg)	29.49 ± 8.51
Lean tissue (kg)	63.58 ± 8.18
Lean Tissue and Bone Mineral Content (kg)	66.12 ± 8.39
Total Mass (kg)	95.61 ± 14.05
Fat (%)	30.44 ± 5.93
Lean (%)	66.88 ± 5.69
Insulin Sensitivity (X10^− 4^ min^− 1^/µU/ml)	2.03 ± 1.47
Acute Insulin Response (µU/ml X 10 min)	1377.75 ± 849.43
Disposition Index (X10^− 4^ min^− 1^)	2158.26 ± 1193.65
Fasting Glucose (mg/dl)	82.38 ± 9.55
CRP (ng/ml)	1958.11 ± 1982.61
Adiponectin (µg/ml)	7.53 ± 2.54

**Fig. 1:**
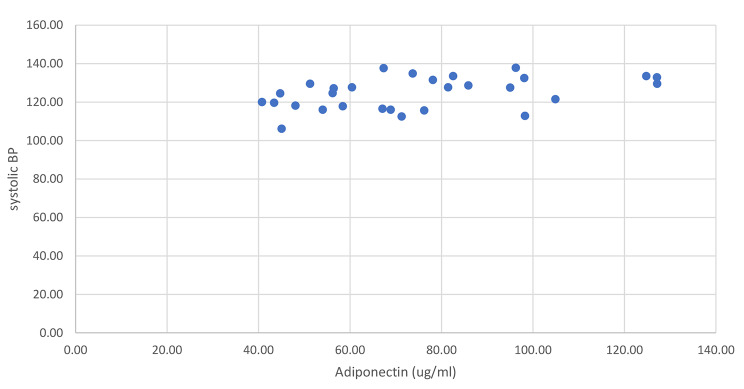
Correlation between Systolic Blood Pressure and Adiponectin

**Fig. 2:**
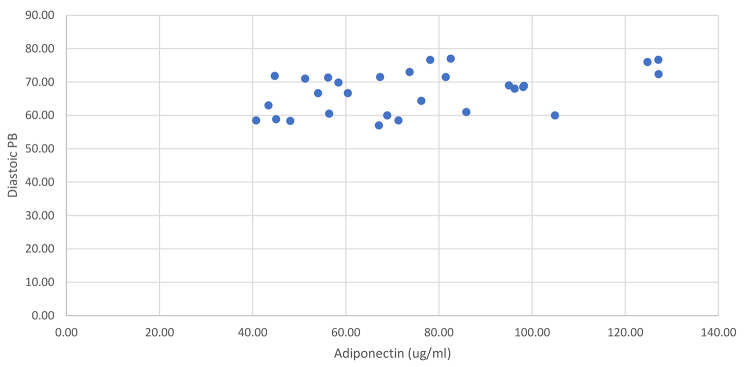
Correlation between Diastolic Blood Pressure and Adiponectin

There were no correlations between SBP and age, height, BMI percentile, waist circumference, hip circumference, waist/hip ratio, fat mass, lean tissue and bone mineral content, disposition index, acute insulin response, and CRP (p > 0.05; see Table 1). Whereas positive nonsignificant relationships were observed between BMI and Weight, pulse pressure, lean tissue, total mass, % fat, % lean, insulin sensitivity, and glucose (p > 0.05; see Table 1). However, adiponectin, showed a moderate positively significant correlation with SBP (rho = 0.436, p = 0.018, see Fig. 1). For DPB, there were weak positive non-significant relationships with age, weight, BMI, pulse pressure, lean tissue, % lean tissue, SI, AIR, and glucose (p > 0.05; see Table 1). Weak negative non-significant relationships were observed between DPB and height, BMI %tile, waist and hip circumference, waist/hip ratio, fat mass, %fat, DI, and CRP (p > 0.05; see Table 1). There was a positive moderate significant correlation between DPB and adiponectin (rho = 0.41, p = 0.02; see Fig. 2).

A multivariate linear regression analysis was conducted to investigate whether plasma adiponectin levels could significantly detect SBP and DBP. The results of the regression analysis showed that the model with Adiponectin only could significantly detect 19% of the variance in SBP (R^2^ = (0.192) F [1, 25] = 6.437, p = 0. 017; see Table 2). It was also found that plasma adiponectin detects SBP (β = 0.439 ± 0.61, p = 0.022). For DPB, the results of the regression analysis showed that the model with Adiponectin only significantly detected 18% of the variance in DPB (R^2^ = (0.183) F [2, 24] = 5.598, p = 0.026). Plasma adiponectin also detects DPB (β = 0.428 ± 0.047, p = 0.026). Plasma adiponectin detected 8% of the variance of pulse pressure (R^2^ = (0.113) F [1, 25] = 3.441, p = 0. 075). However, plasma adiponectin did not significantly detect pulse pressure (β = 0.199 ± 0.107, p = 0.75). A simple liner regression did not show a relation between adiponectin and BMI (p < 0.63), fat mass, lean tissue (p = 0.57), waist circumference (p = 0.164), fasting glucose (p = 0.396), SI (p = 0.558), DI (p = 0.423), or AIR (p = 0.944).

## Discussion

To the best of our knowledge this is the first study to investigate the relationship between plasma adiponectin and blood pressure in Latino adolescent males with obesity with a family history of type 2 diabetes. Our results show a significant positive relationship between plasma adiponectin and blood pressure, with adiponectin significantly detecting blood pressure in obese Latino adolescent males. Furthermore, our results did not show a relation between adiponectin and other study variables. Many adult and pediatric studies have demonstrated a clear relationship between plasma adiponectin and most variables of the metabolic syndrome [26, 27]. Several physiological processes have been proposed to explain the relationship this relationship including (1) enhanced endothelial function and anti-inflammatory macrophage phenotypes, (2) increased nitric oxide production, and (3) in addition to suppressing sympathetic nervous system activity and reduce blood pressure by induce adiponectin secretion [19]. However, the relationship between plasma adiponectin and blood pressure is less definitive and can be quite contradictory.

A few studies in adults have also shown a significant relationship between adiponectin and blood pressure [28, 30, 31]. In Pediatrics, data has shown an inverse relationship between adiponectin and blood pressure [32, 34], these results are conflicting to those presented in this study, which found a positive relationship between these variables. Most of these pediatric studies investigated this relationship in non-obese, non-Latino [35, 36], a small number were also in obese children [37, 39], but none investigated this relationship in pediatric obese Latino males with a family history of type 2 diabetes. A report by Huang et al. showed an inverse relationship between SBP blood pressure but not DBP in a 68 non obese, nondiabetic females. The authors concluded that was independent of any other anthropometric and metabolic variable [36].

Shatat et al. also demonstrated in 41 obese and non-obese adolescents with and without the presence of Type 2 Diabetes that adiponectin levels were independently and inversely associated with 24-hr SBP and DBP [39]. There results also showed no significant differences in adiponectin by gender, with the authors speculating the results could be attributed to the morbid obesity and its independent effect on reduced adiponectin levels. Because our participants were obese, fat distribution influenced adiponectin secretion. Shatat et al. study did have a similar sample size of 26 participants to the present study, their participants were male and females, some of their obese participants also were prehypertensive and hypertensive and included 10 black adolescent participants. Furthermore, pathological states such as metabolic syndrome and obesity have been shown to have an association with higher sympathetic nervous system activity. It may be possible that their results differed from ours for these reasons. Preliminary data of more than 100 male adolescents reported by Hunang et al., showed no relationship between adiponectin and BP [36]. It is also possible that the relationship between adiponectin and BP may vary by race [40], thus explaining the differences in results from our study with that of Shat et al. African American [40, 41] and Asian Indians have been shown to have lower adiponectin levels when compared to white [42]. Zhou et al. investigated the relationship between plasma adiponectin and blood pressure in a very large sample 1300 of children aged 9 to 16 years also found no significant associations [43]. In contrast, Mallamaci et al. found in a sample of 36 hypertensive and 31 normotensive adults found similar results to our study in that they too found a positive association between plasma adiponectin and blood pressure [44]. These and our results suggest that the association between adiponectin and blood pressure may be placing these obese boys with a family history of type 2 diabetes at risk for said disease along with other metabolic and cardiovascular events [45, 46] and an increased risk of future heart failure [47].

There are sever limitations to our study worth noting, firstly, the small sample size, we conducted many correlations in our analysis and also we did not find a correlation between age or height and blood pressure, this was probably due to our study being underpowered to detect correlations; 2) this is a cross -sectional study design, which is not an appropriate design to assess cause and effect between adiponectin and blood pressure. However, the strength of our study is (1) the homogeneous sample, (2) the precise techniques; (3) representative sample of the obese Latino adolescent community and (4) as gender differences were not observed in several studies, we chose to focus on males so as not to confound the analysis.

In conclusion, adiponectin and blood pressure are closely related in these adolescent males with obesity and a family history of type 2 diabetes. Future larger studies in the Latino adolescent population need to be conducted due to the biological importance of adiponectin.

## Data Availability

The data for this study is available upon written request. For data requests please contact Dr. Louise Kelly, lakelly@callutheran.edu.

## Abbreviations

BP: Blood Pressure
SBP: Systolic Blood Pressure
DBP: Diastolic Blood pressure

## Declarations

### Clinical Trail Registration Number

NCT01441323.

## References

[1] WHO. Childhood overweight and obesity. 2019. Available from: https://www.who.int/dietphysicalactivity/childhood/en/.

[2] Wang Y, Lobstein T (2006). Worldwide trends in childhood overweight and obesity. Int J Pediatr Obes.

[3] Isasi CR, Parrinello CM, Ayala GX, Delamater AM, Perreira KM, Daviglus ML, Elder JP, Marchante AN, Bangdiwals SI, Van Horn L, Carnethon MR (2016). Sex differences in cardiometabolic risk factors among Hispanic/Latino youth. J Pediatr.

[4] Hu K, Staiano AE. Trends in obesity prevalence among children and adolescents aged 2 to 19 years in the US from 2011 to 2020. JAMA Pediatr. 2022. 10.1001/jamapediatrics.2022.2052 PMC9315946 35877133

[5] Alberti G, Zimmet P, Shaw J, Bloomgarden Z, Kaufman F, Silink M (2004). Type 2 diabetes in the young: the evolving epidemic: the international diabetes federation consensus workshop. Diab Care.

[6] Engin A. The Definition and Prevalence of Obesity and Metabolic Syndrome. Adv Exp Med Biol., 2017; 960:1-17 10.1007/978-3-319-48382-5_1 28585193

[7] Di Sessa A, Umano GR, Miraglia Del Giudice, Santoro E. N. From the liver to the heart: cardiac dysfunction in obese children with non-alcoholic fatty liver disease. World J Hepatol. 2017;9(2):69–73. 10.4254/wjh.v9.i2.69 PMC5241530 28144387

[8] Freedman DS, Mei Z, Srinivasan SR, Berenson GS, Dietz WH (2007). Cardiovascular risk factors and excess adiposity among overweight children and adolescents: the Bogalusa heart study. J Pediatr.

[9] International Agency for Research on Cancer World Health Organization Working Group on the Evaluation of Cancer-Preventive Strategies. Lyon, France, IARC Press, 2002.

[10] Daniels SR (2009). Complications of obesity in children and adolescents. Int J Obes (Lond).

[11] Ghoshal K, Bhattacharyya M, Adiponectin (2015). Probe of the molecular paradigm associating diabetes and obesity. World J Diabetes.

[12] Sorof J, Daniels S (2002). Obesity hypertension in children: a problem of epidemic proportions. Hypertension.

[13] Cruz ML, Huang TT, Johnson MS, Gower BA, Goran MI (2002). Insulin sensitivity and blood pressure in black and white children. Hypertension.

[14] Defronzo RA (1997). Pathogenesis of type 2 diabetes: metabolic and molecular implications for identifying diabetes genes. Diabetes Reviews.

[15] Wang F, Han L, Hu D (2017). Fasting insulin, insulin resistance and risk of hypertension in the general population: a meta-analysis. Clin Chim Acta.

[16] Lona G, Hauser C, Köchli S (2022). Association of blood pressure, obesity, and physical activity with arterial stiffness in children: a systematic review and meta-analysis. Pediatr Res.

[17] Hubert HB, Feinleib M, McNamara PM, Castelli WP (1983). Obesity as an independent risk factor for cardiovascular disease: a 26-year follow-up of participants in the Framingham Heart Study. Circulation.

[18] Cheung EL, Bell CS, Samuel JP, Poffenbarger T, Redwine KM, Samuels JA (2017). Race and obesity in adolescent hypertension. Pediatrics.

[19] Kim DH, Kim C, Ding EL, Townsend MK, Lipsitz LA (2013). Adiponectin levels and the risk of hypertension: a systematic review and meta-analysis. Hypertension.

[20] Yiannikouris F, Gupte M, Putnam K, Cassis L (2010). Adipokines and blood pressure control. Curr Opin Nephrol Hypertens.

[21] Bottner A, Kratzsch J, Muller G (2004). Gender differences of adiponectin levels develop during the progression of puberty 10. And are related to serum androgen levels. J Clin Endocrinol Metab.

[22] Cruz M, Garcia-Macedo R, Garcia-Valerio Y, Gutierrez M, Medina-Navarro R, Duran G, Wacher N, Kumate J (2004). Low adiponectin levels detect type 2 diabetes in mexican children. Diabetes Care.

[23] Shaibi GQ, Cruz ML, Weigensberg MJ, Toledo-Corral CM, Lane CJ, Kelly LA, Davis JN, Koebnick C, Ventura EE, Roberts CK, Goran MI (2007). Adiponectin independently detects metabolic syndrome in overweight latino youth. J Clin Endocrinol Metab.

[24] The fourth report on the (2004). Diagnosis, evaluation, and treatment of high blood pressure in children and adolescents. Pediatr Aug.

[25] Kelly LA, Loza A, Lin X, Schroeder ET, Hughes A, Kirk A, Knowles AM (2015). The effect of a home-based strength training program on type 2 diabetes risk in obese latino boys. J Pediatr Endocrinol Metab.

[26] Kelly L, Holmberg PM, Schroeder ET, Loza A, Lin X, Moody A, Hughes A, Gibson AM, Kirk A (2019). Effect of home-based strength training program on IGF-I, IGFBP-1 and IGFBP-3 in obese latino boys participating in a 16-week randomized controlled trial. J Pediatr Endocrinol Metab.

[27] Yang WS, Lee WJ, Funahashi T, Tanaka S, Matsuzawa Y, Chao CL, Chen CL, Tai TY, Chuang LM (2002). Plasma adiponectin levels in overweight and obese Asians. Obes Res.

[28] Li G, Zhong L, Han L, Wang Y, Li B, Wang D, Zhao Y, Li Y, Zhang Q, Qi L, Speakman JR, Willi SM, Li M, Gao S (2022). Genetic variations in adiponectin levels and dietary patterns on metabolic health among children with normal weight versus obesity: the BCAMS study. Int J Obes (Lond).

[29] Kazumi T, Kawaguchi A, Sakai K, Hirano T, Yoshino G (2002). Young men with high-normal blood pressure have lower serum adiponectin, smaller LDL size, and higher elevated heart rate than those with optimal blood pressure. Diabetes Care.

[30] Iwashima Y, Katsuya T, Ishikawa K, Ouchi N, Ohishi M, Sugimoto K, Fu Y, Motone M, Yamamoto K, Matsuo A, Ohashi K, Kihara S, Funahashi T, Rakugi H, Matsuzawa Y, Ogihara T (2004). Hypoadiponectinemia is an independent risk factor for hypertension. Hypertension.

[31] Chow WS, Cheung BM, Tso AW, Xu A, Wat NM, Fong CH, Ong LH, Tam S, Tan KC, Janus ED, Lam TH, Lam KS (2007). Hypoadiponectinemia as a detector for the development of hypertension: a 5-year prospective study. Hypertension.

[32] Li HY, Chiu YF, Hwu CM, Sheu WH, Hung YJ, Fujimoto W, Quertermous T, Curb JD, Tai TY, Chuang LM (2008). The negative correlation between plasma adiponectin and blood pressure depends on obesity: a family-based association study in SAPPHIRe. Am J Hypertens.

[33] Asayama K, Hayashibe H, Dobashi K, Uchida N, Nakane T, Kodera K, Shirahata A, Taniyama M (2003). Decrease in serum adiponectin level due to obesity and visceral fat accumulation in children. Obes Res.

[34] Beauloye V, Zech F, Tran HT, Clapuyt P, Maes M, Brichard SM (2007). Determinants of early atherosclerosis in obese children and adoles- cents. J Clin Endocrinol Metab.

[35] Liu YL, Liang HR, Liu HT, Li SY, Zhou YY, Cheng HL, Zhou LS (2010). Association of serum adiponectin levels with atherosclerosis and metabolic syndrome in obese children. J Pediatr Endocrinol Metab.

[36] Takemoto K, Deckelbaum RJ, Saito I, Likitmaskul S, Morandi A, Pinelli L, Ishii E, Kida K, Abdalla M (2015). Adiponectin/resistin levels and insulin resistance in children: a four country comparison study. Int J Pediatr Endocrinol.

[37] Huang KC, Chen CL, Chuang LM, Ho SR, Tai TY, Yang WS (2003). Plasma adiponectin levels and blood pressures in nondiabetic adolescent females. J Clin Endocrinol Metab.

[38] De Las Heras J, Lee S, Bacha F, Tfayli H, Arslanian S (2011). Cross-sectional association between blood pressure, in vivo insulin sensitivity and adiponectin in overweight adolescents. Horm Res Paediatr.

[39] Cândido APC, Geloneze B, Calixto A, Vasques ACJ, Freitas RN, Freitas SN, Machado-Coelho GLL, Adiponectin (2021). HOMA-Adiponectin, HOMA-IR in children and adolescents: Ouro Preto Study. Indian J Pediatr.

[40] Shatat IF, Freeman KD, Vuguin PM, Dimartino-Nardi JR, Flynn JT (2009). Relationship between adiponectin and ambulatory blood pressure in obese adolescents. Pediatr Res.

[41] Hulver HW, Saleh O, MacDonald KG, Pories WJ, Barakat HA (2004). Ethnic Differences in Adiponectin Levels Metabolism.

[42] Retnakaran R, HanleyAJ, Raif N, ConnellyPW, Sermer M, Zinman B (2004). Hypoadiponectinaemia in south asian women during pregnancy: evidence of ethnic variation in adiponectin concentration Diabet. Med.

[43] Abate N, Chandalia M, Snell PG, Grundy SM (2004). Adipose tissue metabolites and insulin resistance in nondiabetic asian indian men. J Clin Endocrinol Metab.

[44] Zhuo Q, Wang Z-Q, Fu P, Piao JH, Tian Y, Xu J, Yang XG (2010). Association between adiponectin and metabolic syndrome in older adults from major cities of China. Biomed Environ Sci.

[45] Mallamaci F, Zoccali C, Cuzzola F, Tripepi G, Cutrupi S, Parlongo S, Tanaka S, Ouchi N, Kihara S, Funahashi T, Matsuzawa Y (2002). Adiponectin in essential hypertension. J Nephrol.

[46] Kojima S, Funahashi T, Otsuka F, Maruyoshi H, Yamashita T, Kajiwara I (2007). Future adverse cardiac events can be detected by persistently low plasma adiponectin concentrations in men and marked reductions of adiponectin in women after acute myocardial infarction. Atherosclerosis.

[47] Huang SS, Huang PH, Chen YH, Chiang KH, Chen JW, Lin SJ (2010). Association of adiponectin with future cardiovascular events in patients after acute myocardial infarction. J Atheroscler Thromb.

[48] Lindberg S, Jensen JS, Bjerre M, Pedersen SH, Frystyk J, Flyvbjerg A (2014). Cardio-adipose tissue crosstalk: relationship between adiponectin, plasma pro brain natriuretic peptide and incident heart failure. Eur J Heart Fail.

